# Performance of Different Immobilized Lipases in the Syntheses of Short- and Long-Chain Carboxylic Acid Esters by Esterification Reactions in Organic Media

**DOI:** 10.3390/molecules23040766

**Published:** 2018-03-27

**Authors:** Lionete Nunes de Lima, Adriano Aguiar Mendes, Roberto Fernandez-Lafuente, Paulo Waldir Tardioli, Raquel de Lima Camargo Giordano

**Affiliations:** 1 Graduate Program in Chemical Engineering, Department of Chemical Engineering, Federal University of São Carlos, 13565-905 São Carlos, SP, Brazil; lionetenunes@yahoo.com.br (L.N.d.L.); raquel@ufscar.br (R.d.L.C.G.); 2 Institute of Chemistry, Federal University of Alfenas, 37130-001 Alfenas, MG, Brazil; adriano.mendes@unifal-mg.edu.br; 3 Department of Biocatalysts, ICP-CSIC, Campus UAM-CSIC, 28049 Madrid, Spain

**Keywords:** immobilized lipases, esterification, flavor esters, sugar esters

## Abstract

Short-chain alkyl esters and sugar esters are widely used in the food, pharmaceutical and cosmetic industries due to their flavor and emulsifying characteristics, respectively. Both compounds can be synthesized via biocatalysis using lipases. This work aims to compare the performance of commercial lipases covalently attached to dry acrylic beads functionalized with oxirane groups (lipases from *Candida antarctica* type B—IMMCALB-T2-350, *Pseudomonas fluorescens*—IMMAPF-T2-150, and *Thermomyces lanuginosus*—IMMTLL-T2-150) and a home-made biocatalyst (lipase from *Pseudomonas fluorescens* adsorbed onto silica coated with octyl groups, named PFL-octyl-silica) in the syntheses of short- and long-chain carboxylic acid esters. Esters with flavor properties were synthetized by esterification of acetic and butyl acids with several alcohols (e.g., ethanol, 1-butanol, 1-hexanol, and isoamyl alcohol), and sugar esters were synthetized by esterification of oleic and lauric acids with fructose and lactose. All biocatalysts showed similar performance in the syntheses of short-chain alkyl esters, with conversions ranging from 88.9 to 98.4%. However, in the syntheses of sugar esters the performance of PFL-octyl-silica was almost always lower than the commercial IMMCALB-T2-350, whose conversion was up to 96% in the synthesis of fructose oleate. Both biocatalysts showed high operational stability in organic media, thus having great potential for biotransformations.

## 1. Introduction

Many short-chain alkyl esters exhibit flavors similar to those of common fruits [1,2]. Consequently, they are widely used in pharmaceutical, cosmetics and food industries. Even though aromatic esters might be obtained from natural sources, an alternative to the extraction of these compounds is their chemical synthesis by esterification reactions. These can be catalyzed by inorganic catalysts or biocatalysts [3,4]. Inorganic catalysts require high temperatures, hence requiring high energy costs, and present low selectivity, making the downstream process complex [4]. When using biocatalysts, the reaction conditions are milder and the reactions are more selective [4,5]. In addition, esters synthesized through biocatalysis may be considered closed to “identical to natural” [4,6,7] and thus labeled as green products.

Another ester family of great interest is that composed by the sugar fatty acid esters, which are non-ionic surfactants [8]. These substances are biodegradable, with a varied variety of uses in many different economic sectors, e.g., the cosmetic, food, oral-care, detergent and pharmaceutical industries [4,9,10].

The industrial synthesis of sugar fatty acid esters (e.g., long- or medium-chain fatty acids of glucose, fructose or sucrose) is carried out by transesterification of fatty acid methyl esters (more expensive than the fatty acids) using alkaline or metallic catalysts. The reaction requires high temperatures (>100 °C) and the use of reduced pressure in order to remove the methanol released from the activated acyl donor to prevent competition with the hydroxyl groups of the carbohydrates [8]. In addition, the chemical catalysts are mostly alkaline and the reaction often uses dimethylformamide (DMF), pyridine or dimethylsulfoxide (DMSO) that are toxic solvents. This implies high energy costs, the production of undesired by-products and low selectivity [11,12,13,14,15]. Moreover, the most challenging features of this process are the control of the degree of esterification and the position of acylation [8,16], and both are difficult to achieve using alkaline catalysis. Therefore, enzymatic synthesis of sugar esters might be a useful alternative, which is already used in industrial scale for some applications [4]. In these reactions, in order to dissolve the carbohydrate, the use of very polar solvents is required. The commonly used organic solvents, such as DMF or DMSO, are able to dissolve saccharides, but they usually have very negative effects for enzymes and are not compatible for many applications of carbohydrate-derived products [7,17,18]. Hence, the solvent must reconcile carbohydrate solubility and low polarity to reduce the damage of the enzyme. In addition, the solvent must not present reactive hydroxyl groups able to compete with the sugars, carboxylic groups or esters bonds, which could be substrates of the enzyme and reduce the product yield and/or produce side-products. In general, tertiary alcohols meet these requirements, as lipases do not recognize them as substrates [19,20,21]. Other examples of solvents that might be used in such reactions are ionic liquids [7,22,23,24]. Within the biocatalysts which can be used in the synthesis of esters, the most common ones are lipases [3,4,14,18,25,26,27,28,29,30,31,32,33,34,35]. Lipases can catalyze esterification reactions, provided that the reaction occurs at low water content [36]. Lipases present a peculiar catalytic mechanism, the so-called interfacial activation [37,38]. According to this mechanism, in homogenous aqueous media, the lipase molecules are in a conformational equilibrium between a closed form, with the active center inaccessible for the reaction media due to the presence of a polypeptide chain called lid that block it, and the open form, where the lid is displaced and exposes the lipase active center to the medium [39,40,41]. In the presence of any hydrophobic surface, e.g., a hydrophobic support, another open form of the lipase or a hydrophobic protein [40,42,43], the open form of the lipase becomes adsorbed via the hydrophobic area formed by the surrounding of the active center and the internal side of the lid, shifting the conformational equilibrium and fixing the open form of the lipase stabilized versus the surface of the support [44,45,46].

Esterification is a thermodynamically controlled process, where the yields are only defined by the thermodynamics of the process [21,47], the catalyst only defines the velocity and the possibility of the process [48]. To improve the yields, the thermodynamic equilibrium must be shifted. Reducing the water content (even eliminating the produced water) is the usual way to improve the yields. In these esterification reactions, the use of ultrasound irradiation to prevent the formation of water layers on the immobilized enzyme [49,50], the use of very hydrophobic supports (also to avoid the gathering of water in the enzyme surroundings) [51,52] and the use of molecular sieves to eliminate the produced water [7,32,53,54], have all proven to be very useful to shift the equilibrium and prevent biocatalyst inactivation. In the case of sugars, the situation is more complex, as the catalyst may define the position or positions that are acylated, therefore the biocatalyst may also define in a certain sense the yields of the target product. The possibility of acyl migration must be considered, as this may produce the final modification of positions different to the ones modified by the enzyme [51,55,56].

In this work, lipase from *Pseudomonas fluorescens* (PFL), *Candida antarctica* type B (CALB) and *Thermomyces lanuginosus* (TLL) in one of their commercial immobilized forms (covalently attached to an acrylic resin functionalized with oxirane groups) were used as biocatalysts in the syntheses of esters with flavor and surfactant properties. These lipases, specially CALB, have been frequently employed in the synthesis of esters in nonaqueous medium [4,7,8,10,57,58]. PFL immobilized on silica particles coated with octyl groups (octyl-silica) was also used. Hydrophobically coated supports immobilize the lipases very efficiently and enable the one step immobilization, purification and stabilization of the open form of the lipase, due to the interfacial activation of the lipases on the hydrophobic surface of the support [39,44,48]. Among the several pre-existing supports, silica has been reported as a good choice for enzyme immobilization due to some properties, such as thermal and mechanical stabilities, high surface area, high resistance to organic solvents and microbial degradation and nontoxicity [59]. Biocatalysts prepared by immobilization of several lipases onto ocytl-silica has been successfully used in biotransformation reactions in organic media [26,28,59,60,61]. The preparation of octyl-silica and the immobilization of PFL on this support were previously reported by our group and evaluated in the synthesis of ethyl fatty acid esters [60] and fructose fatty acid ester [28]. In this new research effort, we intend to analyze if the immobilization protocol may affect the final properties of immobilized lipases in esterification reactions involving substrates as different as sugars, short alcohols and short or long chain carboxylic acids.

## 2. Results and Discussion

### 2.1. Synthesis of Aroma Esters

Table 1 shows the conversions observed in the syntheses of short-chain carboxylic acid esters by esterification catalyzed by home-made PFL-octyl-silica and commercial immobilized lipases at 37 °C after 24 h of reaction. All biocatalysts showed excellent performance in the synthesis of flavor esters, yielding conversions ranging from 80.3 to 98.4% after 24 h reaction. Similar results have been reported for other reaction systems (reaction time, enzyme and organic solvent) [4]. PFL-octyl-silica, the home made preparation, offered yields over 95% for all alcohols. IMMTLL-T2-150 offered the worst performance when hexanol was the substrate, only giving 80% after 24 h, and just 90% when isoamyl alcohol and ethanol were used. IMMAPF-T2-150 only offered yields under 90% using ethanol, the higher yields were obtained using butanol (with both, acetic and butyric acid). IMMCALB-T2-350 gave the worst results using ethanol (just over 90%). In an esterification, yields should be identical, thus the difference in yields should be related to some kinetic problem that can slow down the reaction rate in some instances. PFL-octyl-silica seems to be the most suitable biocatalyst for these esterifications among the assayed ones.

### 2.2. Operational Stability of the Different Biocatalyst in the Synthesis of Butyl Butyrate

**Figure 1:**
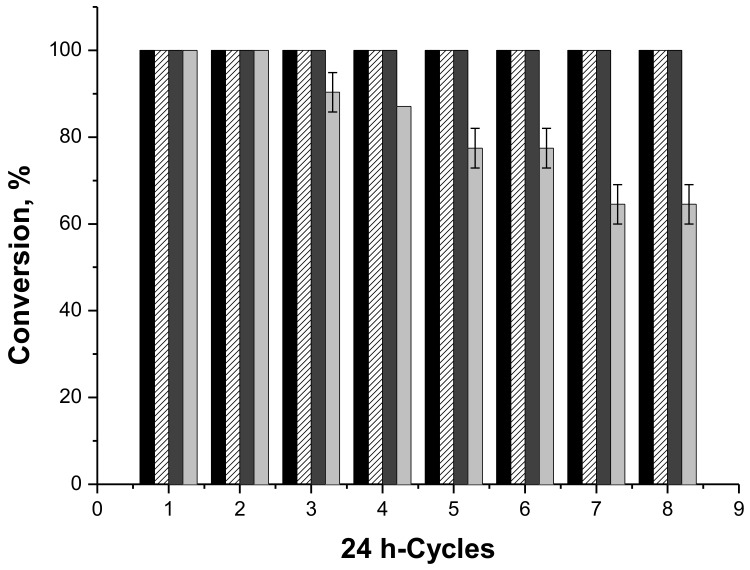
Reaction conversions as function of the number of cycles of butyl butyrate synthesis at 37 °C for 24 h cycles, using 0.1 M in heptane for acids and alcohols, and 200 U/g of acid (esterification activity) and 20 g/L molecular sieves for all biocatalysts. IMMTLL-T2-150 (dark gray column), IMMAPF-T2-150 (gray column), IMMCALB-T2-350 (black column), PFL-octyl-silica (hatched column)-100% was the conversions of the first reaction cycle. All assays were performed in duplicate and the standard deviations were very closed to zero, with only a few closed to 2%.

The operational stabilities of all biocatalysts were evaluated in the syntheses of butyl butyrate at 37 °C for reactions cycles of 24 h (Figure 1). All biocatalysts maintained maximum conversion after eight reaction cycles, except the commercial IMMAPF-T2-150, whose performance in the synthesis of butyl butyrate was greatly reduced to approximately 60% conversion after seven cycles. This contrasts with the very good behavior of PFL-octyl-silica, that suggests a great improvement on the enzyme performance upon immobilization on the hydrophobic support prepared in this work. This could be related to the lower adsorption of water, acids and alcohol in the enzyme environment [51,55,62].

### 2.3. Reaction Course of Butyl Butyrate Synthesis by PFL-Octyl-Silica and IMMCALB-T2-350 Biocatalysts

**Figure 2:**
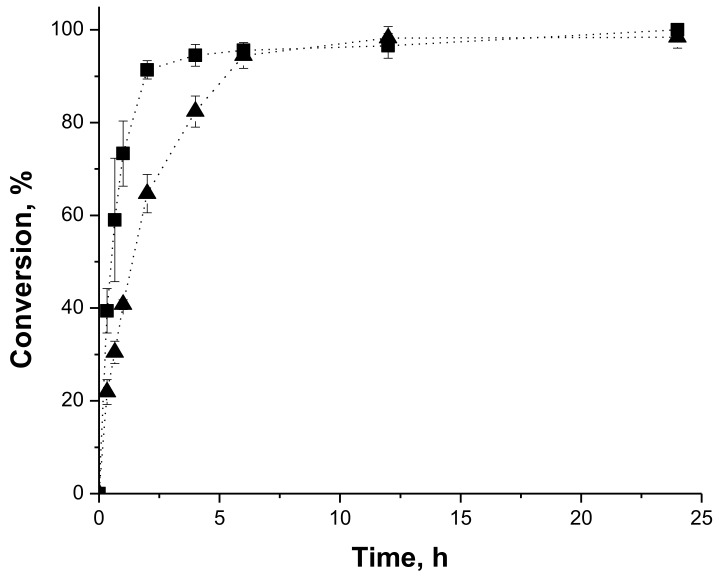
Time-conversion profiles of synthesis of butyl butyrate at 37 °C catalyzed by PFL-octyl-silica in heptane, using 0.1 M for acids and alcohols, 200 U/g of acid (esterification activity) and 20 g/L molecular sieves. The conversions were assessed by consumption of 1-butanol by gas chromatography (squares) and butyric acid by titration (triangles).

**Figure 3:**
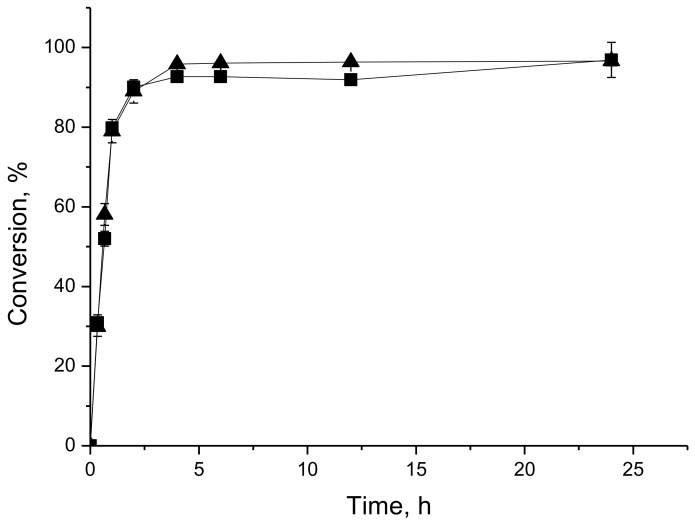
Time-conversion profiles of synthesis of butyl butyrate at 37 °C catalyzed by IMMCALB-T2-350, using 0.1 M in heptane for acids and alcohols, 200 U/g of acid (esterification activity) and 20 g/L molecular sieves. The conversions were assessed by consumption of 1-butanol by gas chromatography (squares) and butyric acid by titration (triangles).

The home-made biocatalyst PFL-octyl-silica and the commercial IMMCALB-T2-350 were chosen to evaluate the reaction course in the conversion of butyl butyrate synthesis. In this set of experiments, the reaction progress was monitored by consumption of both, 1-butanol by gas chromatography and butyric acid by titration. Figure 2 (PFL-octyl-silica catalyzed synthesis) and Figure 3 (IMMCALB-T2-350 catalyzed synthesis) shows that over 90% conversion is achieved within only 2 h of reaction. Although both preparations were very active, IMMCALB-T2-350 was slightly more active in this reaction. Besides, it can be noticed that the conversion calculated from data of titration and gas chromatography agree reasonably well throughout the reaction. Thus, monitoring the reaction by acid titration (particularly when stoichiometric ratio is used) is a simpler and faster method to be used routinely.

### 2.4. Syntheses of Sugar Esters

#### 2.4.1. Syntheses of Fructose Oleate—Stoichiometric Ratio

**Figure 4:**
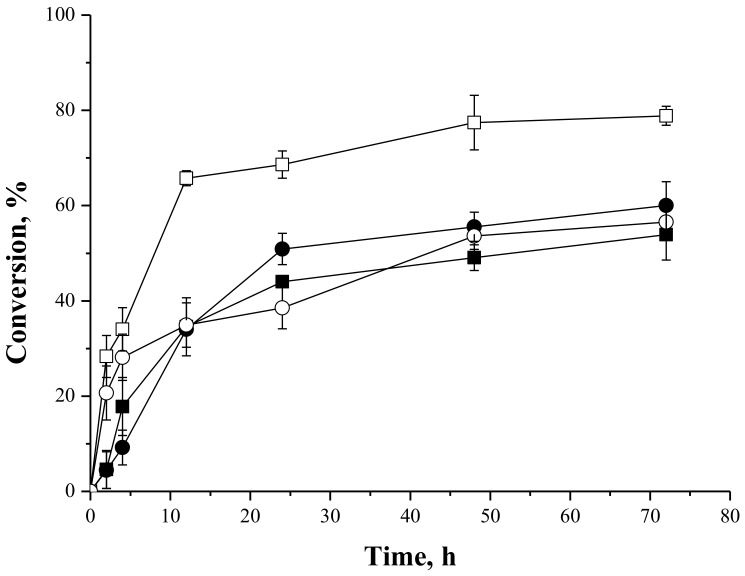
Oleic acid conversion as a function of time for syntheses of fructose oleate at 45 °C, 250 rpm stirring, oleic acid and fructose initial concentrations of 25 mM, using IMMCALB-T2-350 (□) and PFL-octyl-silica (○) in the presence of *tert*-butyl alcohol and IMMCALB-T2-350 (■) and PFL-octyl-silica (●) in the presence of *tert*-amyl alcohol. For all assays 0.5 g of biocatalyst and 1.5 g of molecular sieves were used.

Figure 4 shows reactions courses of syntheses of fructose oleate catalyzed by IMMCALB-T2-350 and PFL-octyl-silica, using *tert*-butyl alcohol or *tert*-amyl alcohol as solvents, which are two of the most frequently employed solvents for lipase-catalyzed carbohydrate ester syntheses [32]. These solvents were chosen because they are sufficiently hydrophobic (logP of 0.80 and 1.4, respectively) to prevent lipase inactivation, but sufficiently hydrophilic to dissolve the carbohydrates [7,32,47]. The highest conversion (79%) was obtained with IMMCALB-T2-350 as biocatalyst and *tert*-butyl alcohol as solvent. This conversion was reduced to 54% when *tert*-amyl alcohol was used as solvent using the same biocatalyst. On the other hand, using PFL-octyl-silica, similar conversions (around 60%) were obtained with both solvents.

It has been reported that interactions between organic solvent molecules and essential water for enzymatic activity in the environment of the enzyme could cause enzyme deactivation, particularly when using more hydrophilic solvents. The more hydrophobic the organic solvent is (higher logP value) the more active and stable the enzyme behaves [32,35,48]. On the other hand, the carbohydrate capability of absorbing water could alter the essential water for the enzyme activity, reducing ester conversion [35]. In these very complex systems, composed by organic solvent, organic acid, the hydrophilic carbohydrate (logP = −2.23, according to the PubChem Data Base), molecular sieves and different support surfaces, it is difficult to explain the behavior of the results with a unique phenomenon. In fact, when using immobilized lipases, the adsorption of hydrophilic substrates (e.g., carboxylic acid and alcohol), water molecules, or reaction products on the support is one of the most relevant cause of biocatalyst inactivation [21].

Neta et al. [63] also reported conversions of 70% and 74% in the syntheses of fructose oleate (stoichiometric ratio of fructose and oleic acid in ethanol as solvent) catalyzed by lipase from *Candida antarctica* B immobilized on chitosan and commercial lipase B from *Candida antarctica*, respectively. However, the authors did not report the selectivity of the reaction, since ethanol can also be a substrate in the esterification reaction. Vescovi et al. [27] reported conversions between 84% and 88% in the synthesis of fructose oleate at 55 °C after 6 h of reaction catalyzed by Novozym 435 (immobilized CALB supplied by Novozymes Latin America Ltda., Araucária, PR, Brazil) and CALB immobilized onto silica bi-functionalized with octyl and glutaraldehyde moieties, using fructose and oleic acid (molar ratio of 1:5) in *tert*-butyl alcohol. Compared to PFL immobilized onto octyl-silica, Vescovi et al. [28] reported conversion around 40% in the synthesis of fructose oleate at 35 °C after 12 h of reaction using fructose and oleic acid (molar ratio of 1:5) in *tert*-butyl alcohol in absence of water. However, when 1% (*v*/*v*) water was added to the reaction medium, around 90% conversion was achieved. These examples show that the reaction conditions (temperature, time reaction, molar ratio of substrates, organic solvent, absence/presence of water, etc.) need to be optimized for each specific biocatalyst.

In our case, PFL-octyl-silica was better catalyst than IMMCALB-T2-350 using *tert*-amyl alcohol as solvent, but it was worse using *tert*-butyl alcohol.

#### 2.4.2. Syntheses of Lactose Oleate—Stoichiometric Ratio

**Figure 5:**
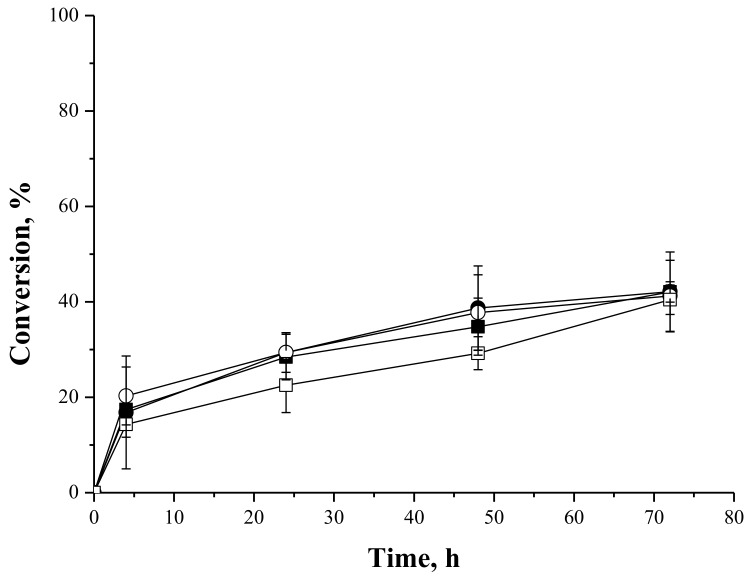
Oleic acid conversion as a function of time for syntheses of lactose oleate at 45 °C, 250 rpm stirring, oleic acid and lactose initial concentrations of 50 mM, using IMMCALB-T2-350 (■) and PFL-octyl-silica (●) in the presence of *tert*-butyl alcohol and IMMCALB-T2-350 (□) and PFL-octyl-silica (○) in the presence of *tert*-amyl alcohol. For all assays 0.5 g of biocatalyst and 1.5 g of molecular sieves were used.

Figure 5 shows the profiles of the reactions courses of the synthesis of lactose oleate catalyzed by IMMCALB-T2-350 and PFL-octyl-silica, using *tert*-butyl alcohol and *tert*-amyl alcohol as solvents. The maximum conversions were around 40% after 72 h reaction using both solvents and biocatalysts. Lactose is a more hydrophilic carbohydrate (logP = −5.03, from the PubChem Data Base) than fructose (logP = −2.23). This higher hydrophilicity of lactose could easily create a layer close to the biocatalyst stripping away essential water for catalytic activity of the enzyme and/or impairing the access of oleic acid to the enzyme active site. Besides, different affinities of lipases for different acyl acceptors in the syntheses of sugar esters have also been reported [64]. Supposedly, more bulky carbohydrate could exhibits more steric hindrances to the formation of the enzyme-substrate complex.

However, high conversion for syntheses of lactose fatty acid esters has been described. Zaidan et al. [65] reported conversions above 70% in the acylation of lactose with capric acid (molar ratio of 2:1) at 55 °C for 48 h, using acetone as solvent and lipase from *Candida rugosa* immobilized on mica with amino groups. Neta et al. [63] also reported conversions above 80% in the synthesis of lactose oleate (40 °C, 72 h reaction, stoichiometric ratio, initial reagent concentration of 83.3 mM) catalyzed by lipase B from *Candida antarctica* immobilized on chitosan and acrylic resin (commercial Novozym 435). However, ethanol was used as solvent and the authors did not report whether ethyl oleate was produced as by-product.

In our case, both solvents and biocatalyst yielded similar yields after 72 h of reaction under the chosen conditions. Curiously, in this reaction IMMCALB-T2-350 performance was not significantly dependent on the solvent used. That is, the binomial solvent/enzyme may change when the substrate changes, showing the complexity of the optimization of these processes.

#### 2.4.3. Syntheses of Fructose and Lactose Laurate—Stoichiometric Ratio

**Figure 6:**
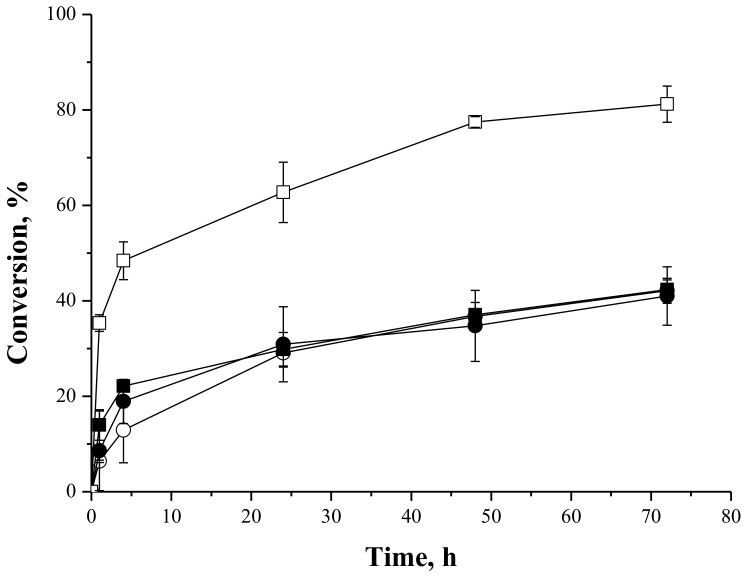
Lauric acid conversion as a function of time for syntheses of fructose laurate at 45 °C, 72 h, 250 rpm, oleic acid and fructose initial concentrations of 25 mM, using IMMCALB-T2-350 (■) and PFL-octyl-silica (●) in the presence of *tert*-butyl alcohol and IMMCALB-T2-350 (□) and PFL-octyl-silica (○) in the presence of *tert*-amyl alcohol. For all assays 0.5 g of biocatalyst and 1.5 g of molecular sieves were used.

**Figure 7:**
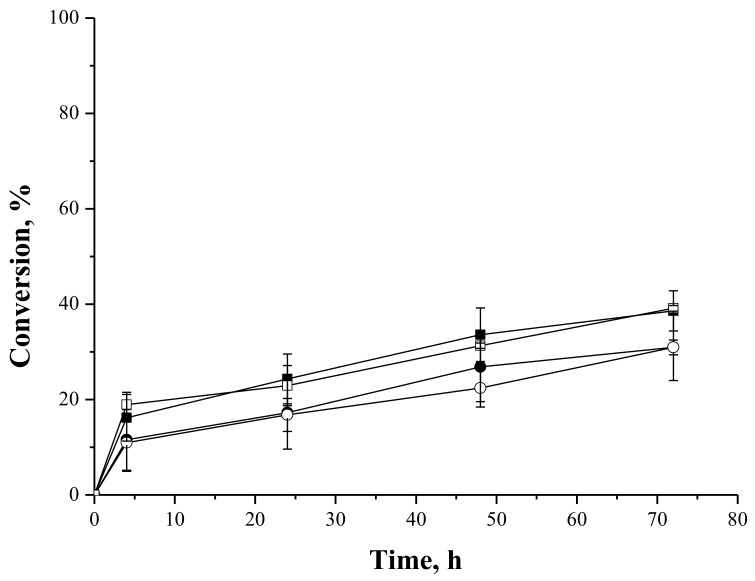
Lauric acid conversion as a function of time for syntheses of lactose laurate at 45 °C, 72 h, 250 rpm stirring, oleic acid and lactose initial concentrations of 50 mM, using IMMCALB-T2-350 (■) and PFL-octyl-silica (●) in the presence of *ter*t-butyl alcohol and IMMCALB-T2-350 (□) and PFL-octyl-silica (○) in the presence of *tert*-amyl alcohol. For all assays 0.5 g of derivative and 1.5 g of molecular sieves were used.

Figure 6 and Figure 7 show the reaction courses of the syntheses of fructose and lactose laurates. Conversions of around 80% were achieved in the acylation of fructose catalyzed by IMMCALB-T2-350 when *tert*-amyl alcohol was used as solvent (Figure 6). Using *tert*-butyl alcohol as solvent, the conversion dropped to 40%. Using PFL-octyl-silica, again there was a lack of effect of the solvent. As pointed above, IMMCALB-T2-350 showed to be more influenced by the solvent.

For the synthesis of lactose laurate (Figure 7), conversions of 40% were achieved using both solvents and IMMCALB-T2-350, while PFL-octyl-silica yields decreased to 30%. This lower enzyme activity versus lactose was observed in the syntheses of lactose oleates (Figure 5). As pointed above, the carbohydrate hydrophilicity and/or the affinity of the lipase seem to be related to the lower conversion using lactose as acyl acceptor. In this case, the effect of the solvent was not relevant for any biocatalyst assayed, suggesting very complex relations and interferences involving, the support, the enzyme and the substrate, as pointed above.

#### 2.4.4. Effect of the Fatty Acid:Carbohydrate Molar Ratio in the Synthesis of Oleate Fructose Esters

**Figure 8:**
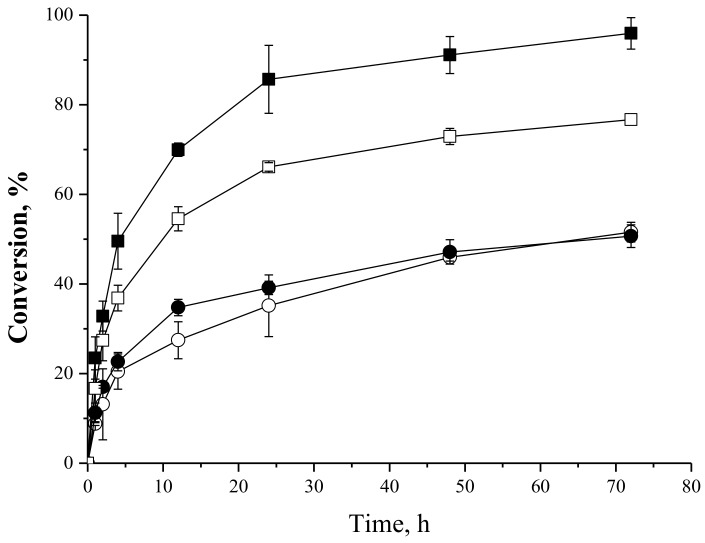
Oleic acid conversion as a function of time for syntheses of fructose oleate in *tert*-butyl alcohol as solvent at 45 °C, 72 h reaction and 250 rpm stirring. Molar ratio oleic acid/fructose of 1:2 (oleic acid initial concentration 25 mM, fructose initial concentration 50 mM) using IMMCALB-T2-350 (■) and PFL-octyl-silica (○). Molar ratio oleic acid/fructose of 2:1 (oleic acid initial concentration 50 mM, fructose initial concentration 25 mM) using IMMCALB-T2-350 (□) and PFL-octyl-silica (●). For all assays 0.5 g of derivative and 1.5 g of molecular sieves were used.

Oleic acid and *tert*-butyl alcohol were chosen for an analysis of the influence of oleic acid:fructose molar ratio on the oleic acid conversion using different biocatalysts. Figure 8 shows the reaction courses for the syntheses of fructose oleate at different concentrations of oleic acid and fructose. It can be observed that the reaction conversion using IMMCALB-T2-350 as biocatalyst yielded 96% when a oleic acid:fructose molar ratio 1:2 (25/50 mM) was used. On the other hand, using PFL-octyl-silica the reaction conversion reached only 50% at the same concentrations of substrates, slightly lower than the one obtained when the oleic acid:fructose molar ratio was 1:1 (25/25 mM), i.e., around 60% conversion. In other words, excess fructose favored the reaction catalyzed by IMMCALB-T2-350, but slightly disfavored the reaction catalyzed by PFL-octyl-silica. The chemical features of the supports are very different and can explain these results. IMMCALB-T2-350 is prepared by covalent linkage of CALB onto an polyacrylic resin activated with oxirane groups (ImmobeadTM IB-350, product catalog from ChiralVision B.V., Leiden, The Netherlands), while PFL-octyl-silica is prepared by hydrophobic adsorption of PFL onto silica surface modified with octyl groups [60]. It has been described that lipase immobilized on hydrophobic supports may be released from the support at presence of organic cosolvents, high temperatures, [66] or in the presence of high concentrations of some substrates-products with surfactant properties [67,68,69]. In the case of PFL-octyl-silica, the reversible nature of the linkage enzyme-support in the presence of the substrate oleic acid and a product with detergent properties (fructose oleate) could desorb the enzyme from the support causing enzyme inactivation, thus reducing the reaction conversion. In fact, as it will be show below, the operational stability of the biocatalyst PFL-octyl-silica was lower than IMMCALB-T2-350 in successive batches of syntheses of fructose oleate.

When excess of oleic acid was used (molar ratio oleic acid:fructose of 2:1) the maximum yield regarding the acid should be 50% if the reaction stopped in monoester. However, reaction conversions higher than 50% were achieved using both biocatalysts, suggesting that some diesters were being produced during the reaction. This is relatively simple to explain manly considering that acyl migrations may liberate the hydroxyl group that is modified by the enzyme [51,55,56].

**Figure 9:**
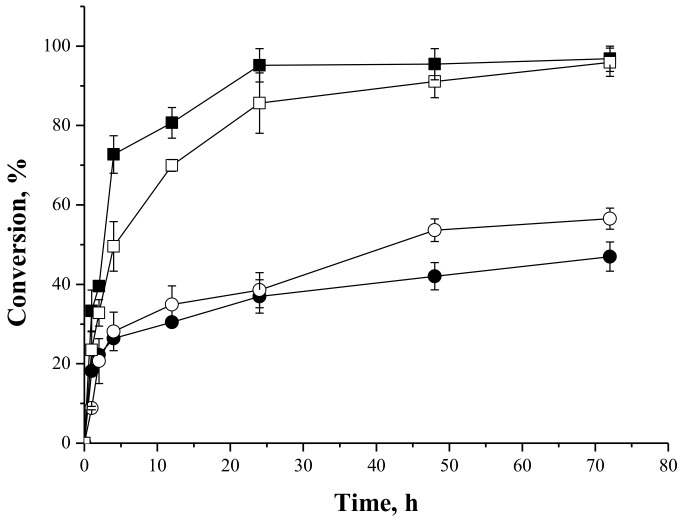
Oleic acid conversion as function of time for syntheses of fructose oleate using *tert*-butyl alcohol as solvent, 72 h reaction, 250 rpm stirring, oleic acid initial concentration 25 mM, fructose initial concentration 25 mM, using PFL-octyl-silica at 45 °C (○), PFL-octyl-silica at 55 °C (●) and oleic acid initial concentration 25 mM, fructose initial concentration 50 mM, using IMMCALB-T2-350 at 45 °C (□) and 55 °C (■). For all assays 0.5 g of derivative and 1.5 g molecular sieves were used.

The effect of the temperature on the conversion of syntheses of fructose oleate was evaluated in the molar ratio oleic acid:fructose of 1:2 (25 mM of oleic acid and 50 mM of fructose) for syntheses catalyzed by IMMCALB-T2-350, and stoichiometric ratio (25 mM of fructose and oleic acid) for syntheses catalyzed by PFL-octyl-silica. Figure 9 shows the reaction courses for syntheses of fructose oleate carried out at 45 °C and 55 °C.

At 55 °C the conversion reached 96% within 24 h using IMMCALB-T2-350 as biocatalyst, while using PFL-octyl-silica the conversion was slightly reduced from 57 (after 72 h at 45 °C) to 46% (after 72 h at 55 °C), although at shorter times the reaction progressed more rapidly at 55 °C. These results could be related to the lower stability of the PFL-octyl-silica due to enzyme desorption from the support surface, followed by enzyme inactivation.

#### 2.4.5. Effect of the Biocatalyst Mass

**Figure 10:**
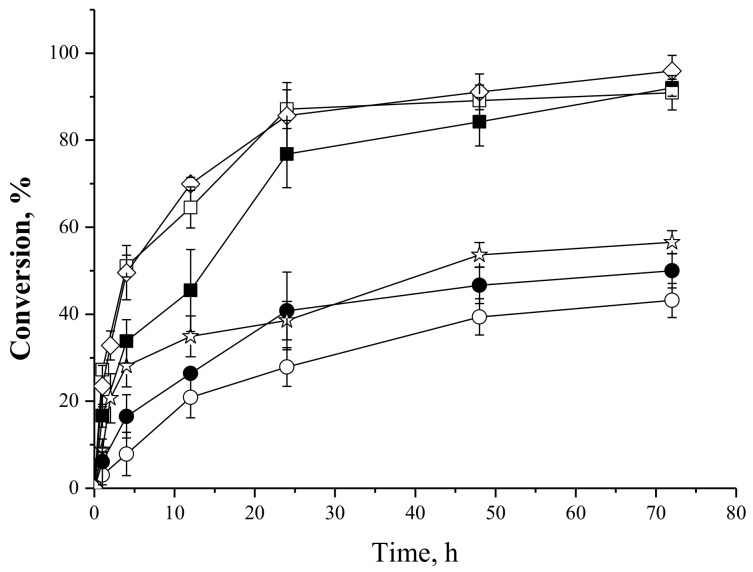
Oleic acid conversion as a function of time on the syntheses of fructose oleate at 45 °C, 250 rpm stirring, fructose initial concentration 25 mM, oleic acid initial concentration 25 mM using 0.25 g (○), 0.5 g (☆) and 1.0 g (●) of PFL-octyl-silica and oleic acid initial concentration 25 mM, fructose initial concentration 50 mM using 0.25 g (■), 0.5 g (◊) and 1.0 g (□) of IMMCALB-T2-350, using *tert*-butyl alcohol as solvent and 1.5 g molecular sieves for all synthesis reactions.

Figure 10 shows the oleic acid consumption during the syntheses of fructose oleate with different derivative masses (0.25, 0.5 and 1.0 g) using initial oleic acid and fructose concentrations of 25 mM when utilizing PFL-octyl-silica as biocatalyst, and oleic acid and fructose initial concentrations of 25 and 50 mM, respectively, when employing IMMCALB-T2-350 as catalyst, using *tert*-butanol as solvent.

For the reactions catalyzed by IMMCALB-T2-350 the behavior was typical of enzyme catalyzed reactions: the initial rate increased with increasing the mass of biocatalyst from 0.25 to 0.5 g, but reaching similar conversions (90 to 96%) for 72 h reaction. When using a higher mass of biocatalyst (1.0 g), the reaction rate did not significantly change, probably because the biocatalyst particles aggregated and produced merged particles with higher diffusional limitations, more water retention, etc. In the reactions catalyzed by PFL-octyl-silica, the reaction rate increased when the biocatalyst mass increased from 0.25 to 0.5 g, but decreased when the mass was increased from 0.5 to 1.0 g, very likely due to the same problem.

#### 2.4.6. Operational Stability of the Biocatalysts in the Syntheses of Fructose Oleate

**Figure 11:**
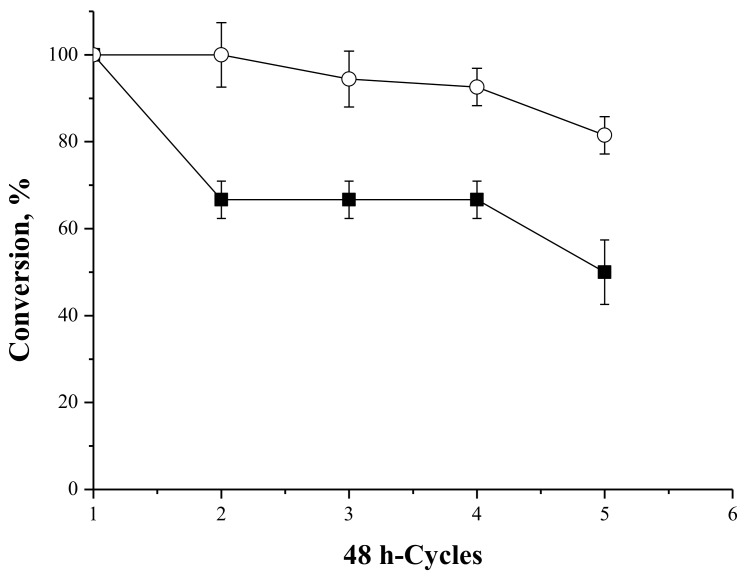
Relative conversion as a function of the number of recycles for syntheses of fructose oleate at 45 °C, cycles of 48 h reaction, 250 rpm stirring, 25 mM of initial concentrations of fructose and oleic acid using PFL-octyl-silica (■) and oleic acid initial concentration of 25 mM, fructose initial concentration of 50 mM using IMMCALB-T2-350 (○). All assays were carried out in *tert*-butyl alcohol as solvent, 0.5 g of biocatalyst and 1.5 g molecular sieves. The conversion for the first cycle was considered as 100%.

Figure 11 shows the operational stability of the biocatalysts PFL-octyl-silica and IMMCALB-T2-350 in successive batches of 48 h in the synthesis of fructose oleate at 45 °C. The performance of both biocatalysts was reduced at 45 °C, but more accentuated for PFL-octyl-silica: the conversion was reduced to 50% after the fifth reaction cycle, while using IMMCALB-T2-350 the conversion was only reduced to 80%. It may be assumed that enzyme desorption from the octyl-silica support occurs in the presence of products with detergent properties (oleic acid and fructose oleate) [67,68,69].

## 3. Materials and Methods

### 3.1. Materials

Lipase from *Pseudomonas fluorescens* (PFL) was acquired from Sigma-Aldrich Co. (St. Louis, MO, USA). Commercial immobilized lipases from *Candida antarctica* type B (CALB, IMMCALB-T2-350), *Thermomyces lanuginosus* (Lipolase, IMMTLL-T2-150) and *Pseudomonas fluorescens* (Amano AK, IMMAPF-T2-150) were acquired from ChiralVision (Leiden, The Netherlands). All these commercial lipases are covalently attached to dry acrylic beads (Immobead^TM^ IB-150 or IB-350—crosslinked copolymer of methacrylate exhibiting oxirane groups—particle size 150–200 micrometer or 300–700 micrometer, respectively) Product catalog (ChiralVision B.V., Leiden, The Netherlands). Macroporous silica (Immobead S60S, particle size of 60–200 micrometer) was attained from ChiralVision (Supplementary material). Molecular sieve UOP type 3 Å (rod, size 1/16 in.) was purposed by Sigma-Aldrich Co. 2-Methyl-2-butanol (*tert*-amyl alcohol), 1-hexanol and 1-butanol were purchased from Sigma-Aldrich Co. Ethanol (99.7%) was purchased from JT Baker (Phillipsburg, NJ, USA). 2-Methyl-2-propanol (*tert*-butyl alcohol), lactose and lauric acid were purchased from Vetec (Rio de Janeiro, Brazil). Heptane, isoamyl alcohol, fructose and oleic acid were purchased from Synth (São Paulo, Brazil). Acetic acid was purchased from Qhemis (Sao Paulo, Brazil).

### 3.2. Production of Octyl-Silica Support

The chemical modification of silica with octyl groups was performed according to Tani and Suzuki [70]. Firstly, silica particles were incubated in 0.1 M hydrochloric acid and washed with distilled water until the pH was neutral. Afterwards, the particles were dried at 200 °C for 5 h. One gram of dried silica was maintained under reflux with 20 mL of a mixture of toluene and octyltriethoxysilane (volume ratio of 10:1) at 85 °C. After 3 h, the suspension was filtered and the support was thoroughly washed with toluene, methanol, and distilled water. Finally, the support was dried at room temperature for 24 h. The activated support, named octyl-silica, was used for the immobilization of PFL.

### 3.3. Immobilization of PFL on Octyl-Silica

Octyl-silica particles were suspended in an enzymatic solution prepared in 5 mM sodium phosphate (pH 7.0) in a support:enzymatic solution ratio of 1:19 (*w*/*v*). Two derivatives were prepared: a low-loaded derivative (5 mg protein/g support) and a high-loaded derivative (40 mg protein/g support), although the loading capability of this support is around 168 mg protein/g support [60]. The suspension was gently stirred in an orbital shaker at 25 °C for 24 h [39]. PFL-octyl-silica biocatalyst was filtered and washed with Milli-Q water. In order to monitor the enzyme immobilization, enzyme hydrolytic activity versus emulsified olive oil was assessed in the supernatant solution and in a control enzyme solution incubated under the same conditions, but without support. The immobilization parameters (immobilization yield and recovered activity) were calculated as previously described [60].

### 3.4. Synthesis of Flavor Esters

The syntheses of flavor esters were performed according to Abbas and Comeau [3] in closed glass flasks. Briefly, 10 mL of a solution of short-chain acids (acetic or butyric acid) and alcohols (1-butanol, 1-hexanol, ethanol or isoamyl alcohol) were prepared in heptane to a final concentration of 0.1 M (for both acid and alcohol). The esterification reactions were performed at 37 °C under 250 rpm stirring in the presence of molecular sieves (20 g/L). A load of 200 U/g of acid (in terms of esterification activity as describe below) was used for all biocatalysts: home-made PFL-octyl-silica (low-loaded derivative, i.e., 5 mg protein/g support) and commercial immobilized lipases (IMMCALB-T2-350, IMMTLL-T2-150 and IMMAPF-T2-150).

The reaction conversion (Equation (1)) was assessed from the results of acid consumption by titration with 0.025 M KOH. Samples of 0.5 mL of the reaction medium were takenat regular time periods and diluted with 10 mL of a solution of acetone:ethanol (1:1 *v*/*v*) containing phenolphthalein as the pH indicator. All assays were performed in triplicate:

(1) $$C\left(\%\right)=\frac{mol_{t=0}-mol_{t=t}}{mol_{t=0}}\times 100$$

where *C*(%) is the reaction conversion, *mol_t_*_=0_ is the initial number of moles of fatty acid and *mol_t=t_* is the number of moles of fatty acid at time *t*.

In order to evaluate whether the titration was suitable to monitor the reaction, the synthesis of butyl butyrate was also followed by gas chromatography. Samples of 1 μL were injected in a HP5890 Gas Chromatograph (Hewlett-Packard Company, Palo Alto, CA, USA) equipped with a flame ionization detector, using nitrogen as carrier gas and a crossbond acid deactivated Carbowax polyethelene glycol column (15 m × 0.32 mm × 0.45 m, Restek Corporation, (Bellefonte, PA, USA)). In this case, the reaction conversion was calculated based on the 1-butanol consumption (in this case, all experiments were carried out using molar ratio butyric acid/1-butanol of 1:1).

### 3.5. Synthesis of Sugar Esters

The syntheses of sugar esters were carried out according to Paula et al. [71] in closed glass flasks. Briefly, 10 mL of a solution of fructose (25 or 50 mM) or lactose (50 mM) were prepared by dissolving the carbohydrates in organic solvents (*tert*-butyl alcohol or *tert*-amyl alcohol). Afterwards, oleic or lauric acids were added to final concentrations of 25 or 50 mM. The reactions were carried out in the presence of 1.5 g of molecular sieves (150 g/L), in order to reduce and control the water activity in the reaction medium. Adachi and Kobayashi [47] reported an increase in the equilibrium conversion for lauroyl mannose synthesis adding to the system until 100 g/L of molecular sieve 3A. In this work, we adopt a concentration of 150 g/L to guarantee water removal from the reaction system. The biocatalysts used in these reactions were PFL-octyl-silica (high-loaded, i.e., 40 mg protein/g support) and the commercial immobilized lipase from *Candida antarctica* (IMMCALB-T2-350), using biocatalyst masses of 0.25, 0.5 and 1.0 g. The reactions were carried out in triplicate at 45 or 55 °C for 72 h under 250 rpm stirring. The reaction conversion was monitored by titration with 0.01 M KOH solution.

### 3.6. Operational Stability of the Biocatalysts

The operational stability of the biocatalysts was studied in the syntheses of butyl butyrate and fructose oleate, as described below.

#### 3.6.1. Flavor Ester (Butyl Butyrate)

PFL-octyl-silica, IMMCALB-T2-350, IMMTLL-T2-150 and IMMAPF-T2-150 were used in eight 24 h-cycles of synthesis of butyl butyrate at 37 °C under 250 rpm stirring, using heptane as solvent, acid and alcohol concentrations of 0.1 M and 20 g/L molecular sieve. The reaction volume and the enzyme load were 10 mL and 200 U/g butyric acid (in terms of esterification activity as described below), respectively. After each cycle, the biocatalysts were washed with heptane before being used in a new reaction cycle. The conversion of the reaction after 24 h was calculated from the consumption of acid, monitored by titration. All assays were performed in duplicate.

#### 3.6.2. Sugar Ester (Fructose Oleate)

PFL-octyl-silica and IMMCALB-T2-350 were employed in five 48 h-cycles of synthesis of fructose oleate employing 0.5 g of biocatalyst, 10 mL of *tert*-butyl alcohol as solvent, and 1.5 g of molecular sieves at 45 °C under 250 rpm stirring. The initial molar ratio fructose/oleic acid was 25:25 (mM) for PFL-octyl silica and 25:50 (mM) for IMMCALB-T2-350. After each 48 h-batch, the biocatalysts were washed with *tert*-butanol and used in a new esterification. The conversion of the reaction was calculated from the consumption of acid, monitored by titration. All assays were carried out in triplicate.

### 3.7. Esterification Activity Assay

The esterification activity of the biocatalysts was measured at 37 °C under 250 rpm stirring in closed glass flasks, using 1-butanol and butyric acid (both at 0.1 M) in heptane (10 mL reaction volume) as acceptor and donor of acyl group, respectively [71]. Samples were taken from the reaction medium at the defined times (up to 40 min) for quantification of butyric acid by titration with 0.0105 M KOH solution. One unit of esterification activity was defined as the consumption of 1 µmol of butyric acid per minute under the reaction conditions.

## 4. Conclusions

Lipase from *Pseudomonas fluorescens* immobilized on silica modified with octyl groups (PFL-octyl-silica biocatalyst) presented similar performance and operational stability compared to the covalent commercial preparation of the same lipase or other lipases in the ester syntheses with flavor properties. On the other hand, different interactions between biocatalysts/medium/substrate might have caused different catalytic behavior of the enzyme during sugar ester synthesis. Such differences might be related either to the nature of the enzymes or interactions between substrates and the biocatalyst itself (support and/or enzyme). In the syntheses of sugar fatty acid esters, PFL-octyl-silica reached lower conversion and showed lower operational stability compared to the CALB IM biocatalyst.

## Figures and Tables

**Table 1:** Conversions of syntheses of short-chain carboxylic acid esters at 37 °C for 24 h, catalyzed by home-made PFL-octyl-silica and commercial immobilized lipases. Reactant concentrations: 0.1 M in heptane for acids and alcohols, and 200 U/g of acid (esterification activity) 20 g/L molecular sieves for all biocatalysts.

Reactants and Products				Conversions (%) by Biocatalyst			
Acyl donor	Acyl acceptor	Ester	FEMA flavor profile ^1^	PFL-octyl-silica	IMMTLL-T2-150	IMMAPF-T2-150	IMMCALB-T2-350
Acetic acid	1-butanol	Butyl acetate	apple, banana, pungent	96.8 ± 0.1	97.0 ± 0.0	93.8 ± 0.4	96.8 ± 0.1
Acetic acid	1-hexanol	Hexyl acetate	apple, banana, grass, herb, pear	98.0 ± 2.9	80.3 ± 1.5	96.9 ± 0.1	97.5 ± 3.5
Acetic acid	isoamyl alcohol	Isoamyl acetate	Apple, banana, glue, pear	96.9 ± 0.2	91.5 ± 0.0	93.7 ± 0.2	95.0 ± 2.4
Butyric acid	1-butanol	Butyl butyrate	floral	98.4 ± 2.3	93.5 ± 0.0	96.9 ± 0.1	96.6 ± 0.1
Acetic acid	ethanol	Ethyl acetate	Aromatic, brandy, grape	96.7 ± 0.1	90.8 ± 0.2	88.9 ± 1.9	90.5 ± 0.3

^1^ Flavor & Extract Manufactures Association (FEMA), available in https://www.femaflavor.org/flavor-library.

## Supplementary Materials

The following are available online.
