# Supplementary material for: Performance of Different Immobilized Lipases in the Syntheses of Short- and Long-Chain Carboxylic Acid Esters by Esterification Reactions in Organic Media
Source: Molecules. 2018 Mar 27;23(4):766. doi: 10.3390/molecules23040766 (PMC6017531; doi:10.3390/molecules23040766)
Supplement: Supplementary file 1 [file molecules-23-00766-s001.pdf]

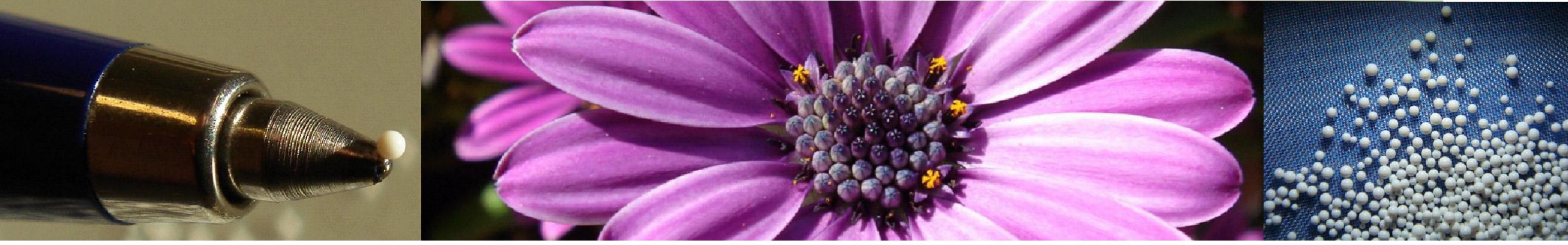

immobilized enzymes - custom immobilization - enzymatic process development – chiral compounds

ChiralVision

innovative biocatalysis!

Product catalog 2013

Immozymes, enzymes & Immobeads

Introduction

ChiralVision offers a range of immobilized enzymes under the brand name Immozymes^{TM}. Immobilization offers easier separation and reuse of enzymes thereby making production processes more robust and cost effective. Additionally, process conditions can be chosen with increased flexibility.

A large collection of different carrier materials in varying particle sizes are screened for applicability to various enzymes. For each individual enzyme optimization of activity and stability is performed. After immobilization all Immozymes are washed extensively. Finally, they are dried yielding an enzyme that can be stored for prolonged times and used in water-free media.

Application tabel

	**Immozyme formulation**			**Recommended medium**		
	absorbed	*covalent*	*dry*	*aqueous*	*biphasic*	*organic*
**T1**	+		+			+
**T2-150**		+	+	+	+	+
**T2-250**		+	+	+	+	+
**T2-350**		+	+	+	++	+++

- particle sizes (availability varies per enzyme):
  - 150 = 150-300 µm
  - 250 = 250-400 µm
  - 350 = 300-700 µm

Special enzymes

We offer special enzymes for asymmetric synthesis comprising ketoreductases, transaminases, aldolases and more**.**

Kits

For screening purposes, all (immobilized) enzymes are available as kits for easy process development.

Immobeads

Immobilization of enzymes and proteins in general required special carriers. ChiralVision offers special carriers with high porosity and various binding modes for optimal immobilization.

Index

Introduction 1

Immozymes 3

Lipases - Immobilized formulations 3

Proteases - Immobilized formulation... 8

Lipases – non-immobilized 11

Genencor proteases.. 12

Kits - overview... 14

Immobeads 16

Immobead – various binding methods 17

Immobead – covalent binding 18

Immobead – ionic binding 19

Immobead – absorption 19

Services... 20

Ordering information 21

Immozymes

Lipases - Immobilized formulations

T1 = absorbed & dry -- T2 = covalent & dry

particle sizes: 150 = 150-300 µm ; 250 = 250-400 µm ; 350 = 300-700 µm

Product nr.	Product specifications	**Pricing in euro***<br>*Delivery time: 1 kg 1-5 days; 10 kg 1-3 weeks*
IMMCALA-T1-350	- lipase A from *Candida antarctica* (CaLA, NovoCor AD L[1]) absorbed on dry acrylic beads<br>- Beadsize 300 - 700 micrometer. Activity: 2000 TBU / g,	99 / 5 g; 249 / 100 g; 1045 / 1 kg; 9050 / 10 kg
IMMCALA-T2-150	- lipase A from *Candida antarctica* (CaLA, NovoCor AD L [1]) covalently attached to dry acrylic beads<br>- Beadsize 150 - 300 micrometer. Activity: 3000 TBU / g	99 / 5 g; 249 / 100 g; 1045 / 1 kg; 9750 / 10 kg
IMMCALA-T2-350	- lipase A from *Candida antarctica* (NovoCor AD L, CaLA[1]) covalently attached to dry acrylic beads<br>- Beadsize 300 - 700 micrometer. Activity: 3000 TBU / g	99 / 5 g; 249 / 100 g; 1075 / 1 kg; 9950 / 10 kg

Product nr.	Product specifications	**Pricing in euro***<br>*Delivery time: 1 kg 1-5 days; 10 kg 1-3 weeks*
IMMCALB-T1-350	- lipase B from *Candida antarctica* (CaLB[1]) absorbed on dry acrylic beads<br>- Beadsize 300 - 700 micrometer. Activity: 4000 TBU / g, 10000 PLU / g	99 / 5 g; 249 / 100 g; 1145 / 1 kg; 9750 / 10 kg
IMMCALB-T2-150	- lipase B from *Candida antarctica* (CaLB[1]) covalently attached to dry acrylic beads<br>- Beadsize 150 - 300 micrometer. Activity: 2500 TBU / g, 5000 PLU / g	99 / 5 g; 249 / 100 g; 1150 / 1 kg; 9500 / 10 kg
IMMCALB-T2-150XL	- lipase B from *Candida antarctica* (CaLB[1]) covalently attached to dry acrylic beads<br>- Beadsize 150 - 300 micrometer. Activity: 8000 TBU / g, 15000 PLU / g	99 / 5 g; 349 / 100 g; 1695 / 1 kg; 14500 / 10 kg
IMMCALB- T2-350	- lipase B from *Candida antarctica* (CaLB[1]) covalently attached to dry acrylic beads<br>- Beadsize 300 - 700 micrometer. Activity: 2500 TBU / g, 5000 PLU / g	99 / 5 g; 249 / 100 g; 1295 / 1 kg; 11500 / 10 kg
IMMCALBY-T1-350	- generic lipase B from *Candida antarctica* (CaLB[2]) absorbed on dry acrylic beads<br>- Beadsize 300 – 700 micrometer. Activity: 2500 TBU / g,	99 / 5 g; 249 / 100 g; 1245 / 1 kg; 11450 / 10 kg
IMMCALBY-T2-150	- generic lipase B from *Candida antarctica* (CaLB[2]) covalently attached to dry acrylic beads<br>- Beadsize 150 – 300 micrometer. Activity: 5000 TBU / g , 8000 PLU / g	99 / 5 g; 249 / 100 g; 1245 / 1 kg; 11450 / 10 kg
IMMCALBY-T2-350	- generic lipase B from *Candida antarctica* (CaLB[2]) covalently attached to dry acrylic beads<br>- Beadsize 300 – 700 micrometer. Activity: 5000 TBU / g	99 / 5 g; 249 / 100 g; 1275 / 1 kg; 11750 / 10 kg
IMMRML-T2-150	- lipase from *Rhizomucor miehei* (Lipozyme[1]) covalently attached to dry acrylic beads<br>- Beadsize 150 – 300 micrometer. Activity: 1500 TBU / g	99 / 5 g; 249 / 100 g; 1950 / 1 kg; 10250 / 10 kg
IMMRML-T2-350	- lipase from *Rhizomucor miehei* (Lipozyme[1]) covalently attached to dry acrylic beads<br>- Beadsize 300 – 700 micrometer. Activity: 1000 TBU / g	99 / 5 g; 249 / 100 g; 1950 / 1 kg; 9500 / 10 kg
IMMTLL-T2-150	- lipase from *Thermomyces lanuginosa* (Lipolase[1]) covalently attached to dry acrylic beads<br>- Beadsize 150 – 300 micrometer. Activity: 10000 TBU / g	99 / 5 g; 249 / 100 g; 995 / 1 kg; 9250 / 10 kg

Product nr.	Product specifications	Pricing in euro*<br>*Delivery time: 1 kg 1-5 days; 10 kg 1-3 weeks*
IMMTLL- T2-350	- lipase from *Thermomyces lanuginosa* (Lipolase[1]) covalently attached to dry acrylic beads<br>- Beadsize 300 – 700 micrometer. Activity: 10000 TBU / g	99 / 5 g; 249 / 100 g; 1045 / 1 kg; 9950 / 10 kg
IMMCRL-T2-150	- lipase from Candida rugosa (CRL[3]) covalently attached to dry acrylic beads<br>- Beadsize 150 - 300 micrometer. Activity: 250 TBU / g	99 / 5 g; 295 / 100 g; 1950 / 1 kg; 10250 / 10 kg
IMMCRL-T2-350	- lipase from Candida rugosa (CRL[3]) covalently attached to dry acrylic beads<br>- Beadsize 300 - 700 micrometer. Activity: 250 TBU / g	99 / 5 g; 295 / 100 g; 1950 / 1 kg; 10250 / 10 kg
IMMCCMO-T2-150	- lipase from *Candida cylindracea sp.* (lipase OF[6]) covalently attached to dry acrylic beads<br>- Beadsize 150 - 300 micrometer. Activity: 275 TBU / g	99 / 1 g; **inquire**
IMMABC-T2-150	- lipase from *Pseudomonas cepacia* (Amano PS[4]) covalently attached to dry acrylic beads<br>- Beadsize 150 - 300 micrometer. Activity: 1500 TBU / g	99 / 5 g; 249 / 100 g; 1045 / 1 kg; 10250 / 10 kg
IMMABC-T2-350	- lipase from *Pseudomonas cepacia* (Amano PS[4]) covalently attached to dry acrylic beads<br>- Beadsize 300 - 700 micrometer. Activity: 500 TBU / g	99 / 5 g; 249 / 100 g; 1045 / 1 kg; 10250 / 10 kg
IMMAPF-T2-150	- lipase from *Pseudomonas fluorescens* (Amano AK[4]) covalently attached to dry acrylic beads<br>- Beadsize 150 - 300 micrometer. Activity: 1000 TBU / g	99 / 5 g; 249 / 100 g; 1045 / 1 kg; 10250 / 10 kg
IMMAPF-T2-350	- lipase from *Pseudomonas fluorescens* (Amano AK[4]) covalently attached to dry acrylic beads<br>- Beadsize 300 - 700 micrometer. Activity: 1000 TBU / g	99 / 5 g; 249 / 100 g; 1045 / 1 kg; 10250 / 10 kg
IMMARO-T2-150	- lipase from *Rhizopus oryzae* (Amano F-AP15[4]) covalently attached to dry acrylic beads<br>- Beadsize 150 - 300 micrometer. Activity: 800 TBU / g	99 / 5 g; 249 / 100 g; 1865 / 1 kg; 10250 / 10 kg
IMMARO-T2-350	- lipase from *Rhizopus oryzae* (Amano F-AP15[4]) covalently attached to dry acrylic beads<br>- Beadsize 300 - 700 micrometer. Activity: 600 TBU / g	99 / 5 g; 249 / 100 g; 1865 / 1 kg; 10250 / 10 kg

Product nr.	Product specifications	Pricing in euro*<br>*Delivery time: 1 kg 1-5 days; 10 kg 1-3 weeks*
IMMAMJ-T2-150	- lipase from *Mucor javanicus* (Amano M[4]) covalently attached to dry acrylic beads<br>- Beadsize 150 - 300 micrometer. Activity: 300 TBU / g	99 / 5 g; 249 / 100 g; 1250 / kg
IMMANA-T2-150	- lipase from *Aspergillus niger* (Lipase A[4]) covalently attached to dry acrylic beads<br>- Beadsize 150 - 300 micrometer. Activity: 40 TBU / g	99 / 5 g; 249 / 100 g; 1250 / kg
IMMRNA-T2-150	- lipase from *Rhizopus niveus* (Lipase[4]) covalently attached to dry acrylic beads<br>- Beadsize 150 - 300 micrometer. Activity: 15 TBU / g	99 / 5 g; 249 / 100 g; 1250 / kg
IMMASMQ-T2-150	- lipase from *Alcaligenes sp.* (lipase QLM[6]) covalently attached to dry acrylic beads<br>- Beadsize 150 - 300 micrometer. Activity: 550 TBU / g	99 / 1 g **inquire**
IMMRES-T2-150	- lipase Resinase HT (Resinase HT[1]) covalently attached to dry acrylic beads<br>- Beadsize 150 - 300 micrometer. Activity: 5000 TBU / g	99 / 5 g; 249 / 100 g; 995 / 1 kg; 9250 / 10 kg
IMMLIPX-T2-150	- lipase Lipex 100L (Lipex 100L[1]) covalently attached to dry acrylic beads<br>- Beadsize 150 - 300 micrometer. Activity: 5000 TBU / g	99 / 5 g; 249 / 100 g; 995 / 1 kg; 9250 / 10 kg
IMML51-T1-350	- lipase from *Fusarium solani pisi*, Novozym 51032 (Cutinase[1]) absorbed on dry acrylic beads<br>- Beadsize 300 - 700 micrometer. Activity: 5000 TBU / g,	99 / 5 g; 249 / 100 g; 1050 / 1 kg; 9500 / 10 kg
IML51-T2-150	- lipase from *Fusarium solani pisi*, Novozym 51032 (Cutinase[1]) covalently attached to dry acrylic beads<br>- Beadsize 150 - 300 micrometer. Activity: 5000 TBU / g	99 / 5 g; 249 / 100 g; 995 / 1 kg; 9250 / 10 kg
IMML51-T2-350	- lipase from *Fusarium solani pisi*, Novozym 51032 (Cutinase[1]) covalently attached to dry acrylic beads<br>- Beadsize 300 - 700 micrometer. Activity: 5000 TBU / g	99 / 5 g; 249 / 100 g; 1050 / 1 kg; 9950 / 10 kg
IMMAULI-T1-350	- lipase from *Bacillus subtilis* (lipase[7]) absorbed on dry acrylic beads<br>- Beadsize 300 - 700 micrometer. Activity: 2000 TBU / g	99 / 5 g; 249 / 100 g; 1050 / 1 kg; 9500 / 10 kg

Product nr.	Product specifications	**Pricing in euro*** *Delivery time: 1 kg 1-5 days; 10 kg 1-3 weeks*
IMMAULI-T2-150	- lipase from *Bacillus subtilis* (lipase[7]) covalently attached to dry acrylic beads<br>- Beadsize 150 - 300 micrometer. Activity: 2250 TBU / g	99 / 5 g; 249 / 100 g; 1050 / 1 kg; 9500 / 10 kg
IMMAULI-T2-350	- lipase from *Bacillus subtilis* (lipase[7]) covalently attached to dry acrylic beads<br>- Beadsize 300 - 700 micrometer. Activity: 2250 TBU / g	99 / 5 g; 249 / 100 g; 1050 / 1 kg; 9950 / 10 kg
IMMLIP-1800	- Immozyme lipase Kit, 18 immobilized lipases<br>- covalently attached to dry acrylic beads. Beadsize 150 - 300 micrometer, 1 g each	950 € / kit

Registered trademark from: 1) Novozymes; 2) c-LEcta; 3) Sigma-Aldrich; 4) Amano; 5) Fluka; 6) Meito; 7) Aum Enzymes. All enzymes immobilized by ChiralVision.

Unit definition: 1 TBU unit = 1 μmol butyric acid released per minute / g immobilized enzyme at 40°C and pH 7.5. Download TBU assay.

Unit definition: 1 PLU unit = 1 μmol propyl laurate formed per minute / g immobilized enzyme at 60°C and pH 7.5. Download PLU assay.

*Pricing does not include shipping cost. For bulk pricing contact sales@chiralvision.com, for ordering information [click here]. Contact us for a US dollar quote.

Orders can be send to: sales@chiralvision.com

Proteases - Immobilized formulation

T1 = absorbed & dry -- T2 = covalent & dry -- T3 = covalent & wet

particle sizes: 150 = 150-300 µm ; 350 = 350-700 µm

Product nr.	Product specifications	**Pricing in euro***<br>*Delivery time: 1 kg 1-5 days; 10 kg 1-3 weeks*
IMMALC-T2-250	- protease from *Bacillus sp.*(*Subtilisin,* Alcalase[1]) covalently attached to acrylic beads<br>- Beadsize 250 - 400 micrometer. Activity: 400 ELU / g. (Alcalase liquid = 4000 ELU / ml)	99 / 5 g; 249 / 100 g; 845 / 1 kg; 7990 / 10 kg
IMMSAV-T2-250	- protease from *Bacillus sp.* (*Subtilisin,* Savinase[1]) covalently attached to acrylic beads<br>- Beadsize 250 - 400 micrometer. Activity: 750 ELU / g.	99 / 5 g; 249 / 100 g; 845 / 1 kg; 7990 / 10 kg
IMMEVE-T2-250	- protease from *Bacillus sp.* (*Subtilisin,* Everlase[1]) covalently attached to acrylic beads<br>- Beadsize 250 - 400 micrometer. Activity: 100 ELU / g.	99 / 5 g; 249 / 100 g; 845 / 1 kg; 7990 / 10 kg
IMMESP-T2-250	- protease from *Bacillus sp.* (*Subtilisin,* Esperase[1]) covalently attached to acrylic beads<br>- Beadsize 250 - 400 micrometer. Activity: 275 ELU / g.	99 / 5 g; 249 / 100 g; 845 / 1 kg; 7990 / 10 kg
IMMCAR-T2-150	- protease from *Mucor miehei* (Acid protease[1]) covalently attached to acrylic beads<br>- Beadsize 150 - 300 micrometer. Activity: 8 ELU / g.	99 / 5 g; 249 / 100 g; 845 / 1 kg; 7990 / 10 kg
IMMP6-T2-250	- protease from *Bacillus licheniformis* (Protex 6L, Multifect PR 6L [2]) covalent on acrylic beads<br>- Beadsize 250 - 400 micrometer. Activity: 400 ELU / g. (Protex 6 liquid = 4000 ELU / ml)	99 / 5 g; 249 / 100 g; 845 / 1 kg; 7990 / 10 kg

Product nr.	Product specifications	**Pricing in euro*** *Delivery time: 1 kg 1-5 days; 10 kg 1-3 weeks*
IMMP7-T2-150	- protease from *Bacillus amyloliquefaciens* (Protex 7L, Multifect PR 7L [2]) covalently attached to acrylic beads<br>- Beadsize 150 - 300 micrometer. Activity: 15 ELU / g.	99 / 5 g; 249 / 100 g; 845 / 1 kg; 7990 / 10 kg
IMMP14-T2-150	- protease from *Geobacillus sp. (Thermolysin*, Protex 14L, Multifect PR 14L [2]) covalent on acrylic beads<br>- Beadsize 150 - 300 micrometer. Activity: 5 ELU / g.	99 / 5 g; 249 / 100 g; 845 / 1 kg; 7990 / 10 kg
IMMP15-T2-150	- protease from *Trichoderma reesei* (Protex 15L, Multifect PR 15L [2]) covalent on acrylic beads<br>- Beadsize 150 - 300 micrometer. Activity: 5 ELU / g.	99 / 5 g; 249 / 100 g; 845 / 1 kg; 7990 / 10 kg
IMMP30-T2-150	- protease from *Bacillus subtilis* (Protex 30L, Multifect PR 30L [2]) covalent on acrylic beads<br>- Beadsize 150 - 300 micrometer. Activity: 225 ELU / g.	99 / 5 g; 249 / 100 g; 845 / 1 kg; 7990 / 10 kg
IMMP40L-T2-150	- protease from *Bacillus subtilis* (Protex 40L, OPTIMASE PR 40L [2]) covalent on acrylic beads<br>- Beadsize 150 - 300 micrometer. Activity: 400 ELU / g.	99 / 5 g; 249 / 100 g; 845 / 1 kg; 7990 / 10 kg
IMMP50-T2-150	- protease from *Aspergillus oryzae var.* (Protex 50 FP, Multifect PR 50G [2]) covalent on acrylic beads<br>- Beadsize 150 - 300 micrometer. Activity: 5 ELU / g.	99 / 5 g; 249 / 100 g; 845 / 1 kg; 7990 / 10 kg
IMMP51-T2-150	- protease from *Aspergillus oryzae* (Protex 51 FP, Multifect PR 51G [2]) covalently attached to dry acrylic beads<br>- Beadsize 150 - 300 micrometer. Activity: 65 ELU / g.	99 / 5 g; 249 / 100 g; 845 / 1 kg; 7990 / 10 kg
IMMP89-T2-250	- protease from *Bacillus subtilis* (Protex 89L, OPTIMASE PR 89L [2]) covalently attached to acrylic beads<br>- Beadsize 250 - 400 micrometer. Activity: 150 ELU / g.	99 / 5 g; 249 / 100 g; 845 / 1 kg; 7990 / 10 kg
IMMAUAC-T2-150	- protease from *Aspergillus niger* (Acid protease[3]) covalently attached to acrylic beads<br>- Beadsize 150 - 300 micrometer. Activity: 5 ELU / g.	99 / 5 g; 249 / 100 g; 845 / 1 kg; 7990 / 10 kg

Product nr.	Product specifications	**Pricing in euro*** *Delivery time: 1 kg 1-5 days; 10 kg 1-3 weeks*
IMMAUNE-T2-150	- protease from *Bacillus subtilis* (Neutral protease[3]) covalently attached to acrylic beads<br>- Beadsize 150 - 300 micrometer. Activity: 10 ELU / g.	99 / 5 g; 249 / 100 g; 845 / 1 kg; 7990 / 10 kg
IMMAUAL-T2-250	- protease from *Bacillus subtilis* (Alkaline protease[3]) covalently attached to acrylic beads<br>- Beadsize 250 - 400 micrometer. Activity: 175 ELU / g.	99 / 5 g; 249 / 100 g; 845 / 1 kg; 7990 / 10 kg
IMMPAP-T2-150	- protease from *Carica papaya* (Papain[4]) covalently attached to acrylic beads<br>- Beadsize 150 - 300 micrometer. Activity: 5 ELU / g.	99 / 5 g; 299 / 100 g; 1250 / 1 kg; 8450 / 10 kg
IMMBRO-T2-150	- protease from *pineapple stem* (Bromelain[4]) covalently attached to acrylic beads<br>- Beadsize 150 - 300 micrometer. Activity: 5 ELU / g.	99 / 5 g; 499 / 100 g; 699 / 250 g; 2150 / kg
IMMFIC-T2-150	- protease from *fig tree latex* (Ficin[4]) covalently attached to acrylic beads<br>- Beadsize 150 - 300 micrometer. Activity: 5 ELU / g.	99 / 5 g; 249 / 25 g;
IMMPROT-2000	- Immozyme protease Kit, 20 immobilized proteases covalently attached to acrylic beads<br>- Beadsize 150 - 300 micrometer, 1 gram each	975 /kit

Registered trademark from: 1) Novozymes; 2) Genencor; 3) Aum Enzymes 4) Sigma-Aldrich. All enzymes immobilized by ChiralVision

Unit definition (ethyl lactate hydrolysis): 1 ELU unit = 1 μmol lactic acid released per minute / g immobilized enzyme at 25°C and pH 6.8. Download ELU assay.

*Pricing does not include shipping cost. For bulk pricing contact sales@chiralvision.com, for ordering information [click here]. Contact us for a US dollar quote.

Orders can be send to: sales@chiralvision.com

Lipases – non-immobilized

ChiralVision offers generic Candida antarctica Lipase A (CaLA) and B (CaLB) as well as isoenzyme pure Candida rugosa lipase (CRL-1) produced recombinantly in yeast by c-LEcta. The non-immobilized enzyme is formulated as dry powder.

The enzyme is available in both research (g – kg) and bulk quantities. For an immobilized formulation see the Immobilized Lipases section.

Product nr.	Product specifications	Pricing in euro*<br>*Delivery time: 1 kg 1-5 days; 10 kg 1-3 weeks*
CV-CALAY	- generic lipase A from *Candida antarctica* (CaLA[1]) produced in yeast<br>- Activity: 45000 TBU / g	99 / g; 249 / 5 g; **inquire**
CV-CALBY	- generic lipase B from *Candida antarctica* (CaLB[1]) produced in yeast<br>- Activity: 43000 TBU / g	75 / 10 g; 249 / 100 g; 900 / 1 kg; 8250 / 10 kg
CV-CRL1	- pure *isoenzyme 1* lipase from *Candida rugosa,* (CRL-1[2]) produced in yeast<br>- Activity: 65000 TBU / g	199 / g; 749 / 5 g; **inquire**

*Pricing does not include shipping cost. For bulk pricing contact sales@chiralvision.com, for ordering information [click here]. Contact us for a US dollar quote.

Orders can be send to: sales@chiralvision.com

Genencor proteases

ChiralVision offers 9 bulk proteases developed and produced by Genencor International in research quantities. For bulk quotes, contact us to get in touch with the right Genencor sales representative. An easy to use screening kit (see the "Genencor protease kit" section below) is exclusively available via ChiralVision.

Product nr.	Product specifications	Pricing in euro*<br>*Delivery time: < 1 liter 1-5 days; 10 liter 1-3 weeks*
CV-P6L	- protease from *Bacillus licheniformis* (Protex 6L, Multifect PR 6L)<br>- Liquid formulation. Activity: 2440 ELU / ml.	125 / 250 ml 295 / liter
CV-P7L	- protease from *Bacillus amyloliquefaciens* (Protex 7L, Multifect PR 7L)<br>- Liquid formulation. Activity: 100 ELU / ml.	99 / 50 ml
CV-P14L	- protease from *Geobacillus sp. (Thermolysin*, Protex 14L, Multifect PR 14L)<br>- Liquid formulation. Activity: 5 ELU / ml.	99 / 50 ml
CV-P15L	- protease from *Trichoderma reesei* (Protex 15L, Multifect PR 15L)<br>- Liquid formulation. Activity: 5 ELU / ml.	99 / 50 ml; 125 / 250 ml; 295 / liter
CV-P30L	- protease from *Bacillus subtilis* (Protex 30L, Multifect PR 30L)<br>- Liquid formulation. Activity: 585 ELU / ml.	99 / 50 ml
CV-P40L	- protease from *Bacillus subtilis* (Protex 40L, OPTIMASE PR 40L)<br>- Liquid formulation. Activity: 1630 ELU / ml.	99 / 50 ml
CV-P50FP	- protease from *Aspergillus oryzae var.* (Protex 50 FP, Multifect PR 50G)<br>- Spray dried formulation. Activity: 20 ELU / g.	99 / 5 g; 249 / 50 g;

Product nr.	Product specifications	**Pricing in euro***<br>*Delivery time: < 1 liter 1-5 days; 10 liter 1-3 weeks*
CV-P51FP	- protease from *Aspergillus oryzae* (Protex 51 FP, Multifect PR 51G) [2]<br>- Spray dried formulation. Activity: 570 ELU / g.	99 / 5 g; 249 / 50 g;
CV-P89L	- protease from *Bacillus subtilis* (Protex 89L, OPTIMASE PR 89L)<br>- Liquid formulation. Activity: 435 ELU / ml.	75 / 50 ml; 125 / 250 ml; 295 / liter
CV-GEN-900	- Kit with 9 proteases (non-immobilized) 20 ml or 5 g each	495 / kit

Protex is a registered tradename of Genencor International

*Pricing does not include shipping cost. For bulk pricing contact sales@chiralvision.com, for ordering information [click here]. Contact us for a US dollar quote.
Unit definition (ethyl lactate hydrolysis): 1 ELU unit = 1 μmol lactic acid released per minute / g immobilized enzyme at 30°C and pH 7.0. Download ELU assay.

Orders can be send to: sales@chiralvision.com

Kits - overview

Lipase and Protease Kits

Catalog No.	Product Name	Description	Euro / kit
IMMHYD-3800	Immozyme hydrolase Kit	38 immobilized enzymes: lipases (18) and proteases (20), 1 g each	1825 €
IMMLIP-1800	Immozyme lipase Kit	18 immobilized lipases, 1 g each	950 €
IMMTRANS-1000	Immozyme transesterification Kit	10 immobilized lipases, 1 g each	495 €
IMMPROT-2000	Immozyme protease Kit	20 immobilized proteases, 1 gram each	975 €
CV-GEN-900	Genencor protease kit	9 proteases (non-immobilized), 20 ml or 5 g each	495 €

Special Enzyme Kits

**Please note these prices are indicative and subject to frequent change due to currency exchange fluctuations. Contact us for a quote. The Special Enzyme Kits are delivered prepaid only.*

Catalog #	Product Name	Description	euro/ 50 mg kit*	euro / 1 mg 96-well plate*
any kit	separate enzyme	1 g each	749	
ES-PLE-600	microbial PLE	6 microbial pig liver esterases, 50 mg each	1281	703
ES-KRED-1500	ketoreductase	150 ketoreductases, 50 mg each	24600	2986
ES-DERA-800	aldolase (DERA)	8 2-Deoxy-D-ribose 5-phosphate aldolases, 50 mg each	1595	703
ES-NIT-4000	nitrilase	40 nitrilases, 50 mg each	6929	1432
ES-CYP-500	cytochrome P450 monooxygenase	5 cytochrome P450 monooxygenases, 50 mg each	1118	703
ES-NHT-2400	nitrile hydratase	24 nitrile hydratases, 50 mg each	4219	1432
ES-ATA-3900	ω-transaminase	39 ω-transaminases, 50 mg each	6821	1432
ES-ERED-2300	ene reductase	23 ene reductases, 50 mg each	4110	1432
ES-AOX-500	alcohol oxidase	5 alcohol oxidases, 50 mg each	1118	703
ES-NTR-1200	nitro reductase	12 nitro reductases, 50 mg each	2289	943
ES-AMD-1500	amidase	15 amidases, 50 mg each	2766	1063

Catalog #	Product Name	Description	euro/<br>50 mg kit*	euro / 1 mg 96-well plate*
ES-D-AADH-1100	D-amino acid dehydrogenase	11 D-amino acid dehydrogenase, 50 mg each	2116	703
ES-EH-200	epoxide hydrolase	2 epoxide hydrolases, 50 mg each	620	
ES-FDH-300	formate dehydrogenase	3 formate dehydrogenases, 50 mg each	815	
ES-GDH-400	glucose dehydrogenase	4 glucose dehydrogenases, 50 mg each	966	
ES-HNL-1800	(S)-oxynitrilase	18 (S)-oxynitrilases, 50 mg each	3243	1432 (S+R)
ES-HNL-1100	(R)-oxynitrilase	11 (R)-oxynitrilases, 5 KU or 50 mg each	2116	
ES-LeuDH-101	Leucine dehydrogenase	1 Leucine dehydrogenase, 1 g	749	
ES-LDH-101	D- Lactate dehydrogenase	D-Lactate dehydrogenase, 1 g	749	
ES-NMNAT-101	nicotinamide mononucleotide adenylyltransferase	nicotinamide mononucleotide adenylyltransferase, 1 g	749	
ES-NADK-101	NAD Kinase	NAD Kinase, 1 g	749	

*****_Please note these prices are indicative and subject to frequent change due to currency exchange fluctuations. Contact us for a quote. The Special Enzyme Kits are delivered prepaid only._

Immobead enzyme carrier Kit

Catalog No.	Product Name	Description	Euro / kit
IMMO-10	Carrier Kit	12 enzyme carriers (for covalent, ionic and absorbtion binding of enzymes), 10 g each	299 €
IMMO-100	Carrier Kit	12 enzyme carriers (for covalent, ionic and absorbtion binding of enzymes), 100 g each	899 €

Immobeads

(Resins for enzyme / protein immobilization)

Introduction

ChiralVision offers a range of enzyme carriers under the brand name Immobead™. Immobilization offers easier separation and reuse of enzymes thereby making production processes more robust and cost effective. Additionally, process conditions can be chosen with increased flexibility.

Enzymes can be immobilized in a variety of ways, that is via:

- covalent binding
- absorption
- ionic binding

Enzyme carriers are available in various particle sizes.

Small: 35 = 35 – 70 µm
Medium: 150 = 150-300 µm
Large: 300 = 300-700 µm
Extra large: 1500 = < 1500 µm

Immobead enzyme / protein carrier Kit

Catalog No.	Product Name	Description	Euro / kit
IMMO-10	Carrier Kit	12 enzyme carriers (for covalent, ionic and absorbtion binding of enzymes), 10 g each	299 €
IMMO-100	Carrier Kit	12 enzyme carriers (for covalent, ionic and absorbtion binding of enzymes), 100 g each	899 €

Immobead – various binding methods

These beads are all macroporous specially selected for immobilization of a variety of enzymes. The porous beads are designed to have a low diffusion limitation that allows for the immobilization of enzymes with high specific activities.

**Product:**	**Pricing*:**
Individual items 99 euro / 100 g	
Carrier kit IMMO-10 (all 12 carriers, 10 g each)	299 euro / kit
Carrier kit IMMO-100 (all 12 carriers, 100 g each)	899 euro / kit

** Shipping not included. For ordering information visit the website. Contact us for a US dollar quote.*

	**Product nr.**	**Type**	**Matrix**	**Functional group**	**Particle size (μm)**
1	IB-150P	Covalent	Polyacrylic	epoxide, polar	150-300
2	IB-150A	Covalent	Polyacrylic	epoxide, apolar	150-300
3	IB-D152	Cationic	Polyacrylic	carboxylic acid	350-700
4	IB-C435	Cationic	Polyacrylic	carboxylic acid	350-700
5	IB-A161	Anionic, strong	Polystyrene	quat. Ammon Type	350-700
6	IB-A171	Anionic, strong	Polystyrene	quat. Ammon Type	350-700
7	IB-A369	Anionic, weak	Polystyrene	quat. Ammon Type	350-700
8	IB-EC1	non-ionic	Polyacrylic	carboxylic ester	350-700
9	IB-S861	non-ionic	Polystyrene	aromatic	350-700
10	IB-S500	non-ionic	Polypropylene	alkyl	150-1500
11	IB-S60P	non-ionic	silica, porous	hydroxyl	60-200
12	IB-S60S	non-ionic	silica, super porous	hydroxyl	60-200

Immobead – covalent binding

This bead is a macroporous acrylic polymer carrying epoxide residues suitable for the covalent immobilization of a variety of enzymes. The porous beads are especially designed to have a low diffusion limitation that allows for the immobilization of enzymes with high specific activities like lipases.

IB-150P

- Crosslinked copolymer of methacrylate carrying oxirane groups.
- polar, hydrophilic
- Particle size: 150-300 µm
- Moisture content < 5%

Product nr.	name	kg	price*
IB-150P	Immobead 150P	0.1	99
IB-150P	Immobead 150P	1	360 / kg
IB-150P	Immobead 150P	10	285 / kg
IB-150P	Immobead 150P	*ton scale available*	*contact us*

*Shipping not included

IB-150A

- Crosslinked copolymer of methacrylate carrying oxirane groups.
- apolar, hydrophobic
- Particle size: 150-300 µm
- Moisture content < 5%

Product nr.	name	kg	price
IB-150A	Immobead 150A	0.1	99
IB-150A	Immobead 150A	1	495 / kg
IB-150A	Immobead 150A	10	475 / kg
IB-150A	Immobead 150A	*ton scale available*	*contact us*

*Shipping not included

**For ordering information and shipping costs visit the website.*
Contact us for a US dollar quote.

Orders can be send to: sales@chiralvision.com

Immobead – ionic binding

These beads are macroporous polymers suitable for the immobilization of various types of enzymes. The beads bind enzymes via a positively or a negatively charged group.

Immobead - Ionic

- Crosslinked polyacrylic or polystyrenic polymer
- Particle size: 350-700 µm
- Moisture content 50 - 60%

Product nr.	Type	Matrix	Functional group	price / kg*
IB-D152	Cationic	Polyacrylic	Carboxylic acid	249
IB-C435	Cationic	Polyacrylic	Carboxylic acid	249
IB-A161	Anionic, strong	Polystyrene	Quat. Ammon Type	249
IB-A171	Anionic, strong	Polystyrene	Quat. Ammon Type	249
IB-A369	Anionic, weak	Polystyrene	Quat. Ammon Type	249

*Shipping not included

Immobead – absorption

These beads are macroporous polystyrene polymers suitable for the immobilization of enzymes via absorption.

Immobead – absorption

- Polystyrene, polypropylene or silica
- Particle size: 350-700 µm, moisture content 2 - 65% (IB-EC1)
- Particle size: 350-700 µm, moisture content 35 - 70% (IB-S861)
- Particle size: 150-1500 µm, moisture content 0-1% (IB-S500)
- Particle size: 60-200 µm, moisture content 2% (IB-S60P and IB-S60S)

Product nr.	Type	Matrix	Properties	price / kg*
IB-EC1	non-ionic	Polyacrylic	carboxylic ester	199
IB-S861	non-ionic	Polystyrene	Macroporous	249
IB-S500	non-ionic	Polypropylene	Macroporous	199
IB-S60P	non-ionic	Silica	Macroporous	199
IB-S60S	non-ionic	Silica	Extra macroporous	299

*Shipping not included

Services

ChiralVision offers a range of enzyme related services including:

- custom immobilization
- enzyme screening
- process optimization
- full chiral process design
- scale-up

Custom immobilization

If your enzyme of choice is not on this list, contact us to see if a prototype is available. If the enzyme is commercially available or if you have a proprietary enzyme ask us for our rapid immobilization service.

The immobilization service offers you quickly first prototypes for evaluation. In an additional service - or if you want to improve performance of your existing immobilized enzyme - multiple rounds of optimization will turn the prototype into a robust catalyst.

Chiral process development service

ChiralVision is *the specialist* in enzymatic resolution and has an extended experience in process development and scale-up of enzymatically prepared (chiral) compounds. We select the right enzyme and identify the most critical areas in order to produce robust, scalable and economical processes.

We can develop new and proprietary routes to existing compounds, or take an existing synthesis and develop a scalable process, all with smart use of biocatalysis. By using hydrolytic bulk enzymes enzyme costs per kilogram of end product can be kept to an absolute minimum. Furthermore, because the enzymes used in existing bulk applications a steady supply and the lowest possible price is guaranteed. The target for enantiomeric excess is >99%.

Time lines
Speed is one of the key requirements for safeguarding competitive advantages – both in developing new processes and in market launches. Screening for the best enzyme for a specific conversion and selection of conditions is performed within the time frame of 2-3 weeks; optimization and initial scale-up of an enzyme-catalyzed reaction is performed in 2 weeks.

What do you get?
- all intellectual property rights derived from the study
- samples of products
- weekly updates
- written report stating all enzymes, chemicals, equipment and procedures used.

Ordering information

Orders can be send to:

- email: sales@chiralvision.com (*email ordering preferred*)
- fax: +31 71 332 2125 or +31 84 758 7301

Please provide:

- contact person (+phone number and email address))
- shipping address
- billing address (*email preferred*)
- internal ordering number
- VAT / tax identification number (in EU mandetory, outside EU it will speed up customs handling)

Shipping cost for 1 kg (UPS Express delivery):

Europe (EU)	40 euro
Europe (non-EU)	50 euro
North-America	50 euro
Asia / all others	65 - 85 euro (inquire)

Cost are based on a weight of 1 kg, for heavier shipments add 12 euro / kg.

**** Contact us for a quote in US dollars ****

ChiralVision' general Terms of Sale (see website) apply. Payment terms: 30 days. Prices are subject to change without notice.

Sales and technical support:

ChiralVision
J.H. Oortweg 21
NL-2333CH Leiden
The Netherlands

info@chiralvision.com
Tel: +31 71 332 2124
Mob. +31 64 603 8991
Fax: +31 71 332 2125 OR +31 84 758 7301

www.chiralvision.com
